# Encapsidation of APOBEC3G into HIV-1 virions involves lipid raft association and does not correlate with APOBEC3G oligomerization

**DOI:** 10.1186/1742-4690-6-99

**Published:** 2009-11-03

**Authors:** Mohammad A Khan, Ritu Goila-Gaur, Sandra Kao, Eri Miyagi, Robert C Walker, Klaus Strebel

**Affiliations:** 1 Laboratory of Molecular Microbiology, Viral Biochemistry Section, National Institute of Allergy and Infectious Diseases, National Institutes of Health, Building 4, Room 310, 4 Center Drive, MSC 0460, Bethesda, MD 20892-0460, USA

## Abstract

**Background:**

The cellular cytidine deaminase APOBEC3G (A3G), when incorporated into the human immunodeficiency virus type 1 (HIV-1), renders viral particles non-infectious. We previously observed that mutation of a single cysteine residue of A3G (C100S) inhibited A3G packaging. In addition, several recent studies showed that mutation of tryptophan 127 (W127) and tyrosine 124 (Y124) inhibited A3G encapsidation suggesting that the N-terminal CDA constitutes a viral packaging signal in A3G. It was also reported that W127 and Y124 affect A3G oligomerization.

**Results:**

Here we studied the mechanistic basis of the packaging defect of A3G W127A and Y124A mutants. Interestingly, cell fractionation studies revealed a strong correlation between encapsidation, lipid raft association, and genomic RNA binding of A3G. Surprisingly, the presence of a C-terminal epitope tag affected lipid raft association and encapsidation of the A3G W127A mutant but had no effect on wt A3G encapsidation, lipid raft association, and interaction with viral genomic RNA. Mutation of Y124 abolished A3G encapsidation irrespective of the presence or absence of an epitope tag. Contrasting a recent report, our co-immunoprecipitation studies failed to reveal a correlation between A3G oligomerization and A3G encapsidation. In fact, our W127A and Y124A mutants both retained the ability to oligomerize.

**Conclusion:**

Our results confirm that W127 and Y124 residues in A3G are important for encapsidation into HIV-1 virions and our data establish a novel correlation between genomic RNA binding, lipid raft association, and viral packaging of A3G. In contrast, we were unable to confirm a role of W127 and Y124 in A3G oligomerization and we thus failed to confirm a correlation between A3G oligomerization and virus encapsidation.

## Background

APOBEC3G (A3G) is a cellular cytidine deaminase with potent antiretroviral activity that severely limits replication of *vif*-defective HIV-1 in human cells [1]. A3G is expressed in most if not all natural human HIV-1 target cells; yet HIV-1 efficiently infects humans and has caused a worldwide pandemic. This ability of HIV-1 to infect and replicate in A3G-positive human cells is made possible by the viral accessory protein Vif, which was found to prevent the packaging of A3G into progeny virions. Inhibition of A3G packaging is accomplished either by proteasome-mediated degradation of A3G or by other degradation-independent mechanisms (reviewed in [2]). Inhibition of A3G encapsidation may also require Vif dimerization since peptide antagonists to Vif dimerization blocked A3G packaging without affecting its intracellular stability [3].

The antiviral effect of A3G generally requires encapsidation of the deaminase into viral particles. Interestingly, the antiviral effects of A3G are not limited to HIV-1 but extend to other retroviruses including murine leukemia virus, mouse mammary tumor virus, simian immunodeficiency virus, equine infectious anemia virus, and hepatitis B virus (for review see [2]). Packaging of A3G into such diverse viruses suggests that virus encapsidation is either relatively nonspecific or involves signals shared by these viruses. Interestingly, although A3G selectively targets single stranded DNA for deamination it also binds RNA. RNA binding of A3G has been shown to contribute to virus encapsidation [4-11]. A3G also interacts with the NC component of the viral Gag precursor protein [7,12-21]. This interaction likely also contributes to the packaging of A3G into viral particles. *In vitro *studies using purified recombinant NC and A3G found that the two proteins do not competitively bind RNA but instead form an RNA-protein ternary complex [5].

Several reports have investigated domains in A3G required for packaging into HIV-1 virions. We and others have recently reported that mutations in the A3G catalytic domain 1 (CD1) can impair A3G packaging [21,22]. Characterization of in-frame deletion mutants implicated a linker region located C-terminal to the CD1 domain (residues 121-161) as critical for A3G packaging into HIV-1 virus-like particles [12,20]. These findings were supported by other studies that identified residues 122 to 127 in the linker domain as important for A3G encapsidation [9,23-26]) It is interesting to note that the adjacent D128 plays an important role in the species specific sensitivity of A3G to Vif [27-30]. Thus, the N-terminal linker region appears to be an important contact point for Vif as well as a requirement for A3G encapsidation. However, there is no conclusive evidence that these regions in A3G constitute direct Vif and/or Gag binding sites as of yet. It is equally possible that these regions impose conformational constraints on the protein that indirectly affect A3G encapsidation or modulate binding of Vif to other regions of the protein. In support of the latter possibility, Stenglein et al. have recently found that the W127A mutation has profound effects on A3G's intracellular localization only in conjunction with simultaneous mutation of Y19 [31]. Based on structural predictions, W127 is located at the protein surface [26,31]. and might therefore be available for a variety of functions including protein-protein and protein-nucleic acid interactions. Indeed, the packaging defect of the A3G W127A mutant was explained by an inability of this mutant to interact with 7SL RNA [9,24]. More recently, the packaging defect of W127A and Y124A mutants was correlated with a defect in A3G oligomerization and the authors proposed that RNA-dependent oligomerization of APOBEC3G was required for restricting HIV-1 [32].

Here we further characterized the role of W127 and Y124 for the packaging of A3G into HIV-1 virions and for A3G oligomerization. Consistent with previous reports we found that packaging of A3G-HA was severely affected by the W127A mutation. Similarly, packaging of Myc epitope-tagged A3G-Myc W127A was severely restricted suggesting that the packaging defect imposed by the W127A mutation is not epitope tag specific. Of note, the effect of the W127A mutation on virus encapsidation was much less severe in the context of untagged A3G. This is surprising and implies that the effects of mutations around position W127 are sensitive to and exacerbated by changes at the C-terminus of the protein. In contrast, mutation of Y124A imposed a severe packaging defect irrespective of the presence or absence of an epitope tag. A3G-HA was previously found to associate with cellular raft structures [14]. Interestingly, our results identified a novel correlation between A3G raft association and virus encapsidation. We analyzed a total of nine A3G variants and found that all packaging competent A3G variants associated with lipid rafts while all packaging incompetent A3G variants failed to do so. We further found that all packaging competent A3G variants interacted with genomic viral RNA as well as 7SL RNA while all packaging incompetent variants interacted with 7SL RNA but failed to interact with viral genomic RNA. Finally, all of our A3G variants analyzed in this study retained the ability to oligomerize irrespective of whether the A3G variant was packaging competent or not. Thus, our data clearly establish a positive correlation between packaging competence of A3G and the ability to associate with lipid rafts and to interact with viral genomic RNA. In contrast, our data failed to verify a correlation between A3G oligomerization and packaging competence. Finally, our results suggest that the presence of C-terminal epitope tags in A3G can impose conformational constraints on A3G that appear to be functionally inconsequential in the context of wild type protein but can exacerbate defects induced by changes to other regions of the protein such as mutation of W127.

## Methods

### Plasmids

The *vif*-defective molecular clone pNL4-3Vif(-)[33] was used for the production of virus. Wild type human A3G carrying a C-terminal Myc epitope tag was described previously [34]. For the expression of untagged human A3G, a stop codon was introduced into pcDNA-A3G-Myc by PCR-directed mutagenesis as reported elsewhere [35]. Mutation of tryptophan residue W127 and tyrosine residue Y124 to alanine in Myc-tagged and untagged human A3G was accomplished by PCR-based mutagenesis of pcDNA-APO3G-Myc and pcDNA-APO3G vector, respectively. The presence of the desired mutation was verified by sequence analysis. Both tagged and untagged A3G were detected by the A3G-specific ApoC17 rabbit polyclonal antibody and were distinguishable by their different mobilities in the gel. Plasmids pA3G, pA3G-HA, pA3G W127A and pA3G-HA W127A expressing untagged and C-terminally HA-tagged A3G wt and W127A mutants in the backbone of pCMV4-HA were a gift of Michael Malim [23].

### Tissue culture and transfection

HeLa cells were propagated in Dulbecco's modified Eagle's medium containing 10% fetal bovine serum (FBS). For transfection, HeLa cells were grown in 25 cm^2 ^flasks to about 80% confluence. Cells were transfected using LipofectAMINE PLUS (Invitrogen Crop., Carlsbad CA) following the manufacturer's recommendations. A total of 5 to 6 μg of plasmid DNA per 25 cm^2 ^flasks (~5 × 10^6 ^cells) was used. Total amounts of transfected DNA were kept constant in all samples of any given experiment by adding empty vector DNA (pUC18 or pcDNA3.1(-)MycHis) as appropriate. Unless stated otherwise, cells were harvested 24 h post-transfection.

### Antisera

A3G was identified using a polyclonal rabbit serum against a synthetic peptide comprising the 17 C-terminal residues of A3G (anti-ApoC17; available through the NIH AIDS Research and Reagent Program, Cat # 10082). Serum from an HIV-positive patient (APS) was used to detect HIV-1-specific capsid (CA) proteins. Tubulin was identified using a monoclonal antibody to α-tubulin (Sigma-Aldrich, Inc., St. Louis MO; Cat # T9026). For immunoprecipitation of tagged and untagged A3G, polyclonal ApoC17 antibody was used. Raft associated marker protein caveolin was identified by polyclonal anti-caveolin antibody (BD Bioscience Pharmingen, San Diego CA; Cat # 610060). Transferrin receptor (TfR) was included as a non raft marker protein and was identified using a TfR-specific monoclonal antibody (BD Bioscience Pharmingen, San Diego CA; Cat # 612125).

### Preparation of virus stocks

Virus stocks were prepared by transfection of HeLa cells with appropriate plasmid DNAs of pNL4-3Vif(-) in the presence of tagged and untagged variants of wild type and mutant (W127A, Y124A) A3G as indicated in the text. Virus-containing supernatants were harvested 24 h after transfection. Cellular debris was removed by centrifugation (3 min, 3,000 × g) and clarified supernatants were filtered (0.45 μm) to remove residual cellular contaminants. For determination of viral infectivity, unconcentrated filtered supernatants were used for the infection of LuSIV indicator cells. For immunoblot analysis of viral protein, virus-containing supernatants (7 ml) were concentrated by ultracentrifugation through 4 ml of 20% sucrose in phosphate-buffered saline (PBS) as described previously [34].

### Infectivity assay

To determine viral infectivity, virus stocks were normalized for equal levels of reverse transcriptase activity and used to infect LuSIV cells (5 × 10^5^) in a 24-well plate in a total volume of 1.2 to 1.4 ml. LuSIV cells are derived from CEMx174 cells and contain a luciferase indicator gene under the control of the SIVmac239 long terminal repeat [36]. These cells were obtained from Janice Clements through the NIH AIDS Research and Reference Reagent Program (catalog # 5460) and were maintained in complete RPMI 1640 medium supplemented with 10% FBS and hygromycin B (300 μg/ml). Cells were infected for 24 h at 37°C. Cells were then harvested and lysed in 150 μl of Promega 1× reporter lysis buffer (Promega Crop., Madison WI). To determine the luciferase activity in the lysates, 50 μl of each lysate was combined with luciferase substrate (Promega. Corp., Madison WI) by automatic injection and light emission was measured for 10 seconds at room temperature in a luminometer (Opticomp II; MGM instruments, Hamden CT).

### Immunoblotting

For immunoblot analysis of intracellular proteins, whole-cell lysates were prepared as follows. Cells were washed once with PBS, suspended in PBS (400 μl/10^7 ^cells), and mixed with an equal volume of sample buffer (4% sodium dodecyl sulfate [SDS], 125 mM Tris-HCL, pH 6.8, 10% 2-mercaptoethanol, 10% glycerol and 0.002% bromophenol blue). Proteins were solubilized by boiling for 10 to 15 min at 95°C, with occasional vortexing of the samples to shear cellular DNA. Residual insoluble material was removed by centrifugation (2 min, 15,000 rpm, in an Eppendorf Minifuge). For immunoblot analysis of virus-associated proteins, concentrated viral pellets were suspended in a 1:1 mixture of PBS and sample buffer and boiled. Cell lysates and viral extracts were subjected to SDS-polyacrylamide gel electrophoresis; proteins were transferred to polyvinylidene diflouride membranes and reacted with appropriate antibodies as described in the text. Membranes were then incubated with horseradish peroxidase (HRP)-conjugated secondary antibodies (GE Healthcare Biosciences, Piscataway NJ) and visualized by enhanced chemiluminescence (GE Healthcare Biosciences).

### Immunoprecipitation

For immunoprecipitation of tagged and untagged A3G, A3G W127A, and A3G Y124A, lysates of transfected cells were prepared as follows. Cells were washed once with PBS and lysed in 300 μl of lysis buffer (50 mM Tris, pH 7.5, 150 mM NaCl, 0.5% Triton X-100). Cell extracts were clarified at 13,000 × g for 3 min, and the supernatant was incubated on a rotating wheel for 1 h at 4°C with protein A-Sepharose coupled with anti-ApoC17 antibody. Immune complexes were washed three times with 50 mM Tris, 300 mM NaCl, and 0.1% Triton X-100, pH 7.4. Bound proteins were eluted from beads by heating in sample buffer for 5 min at 96°C and analyzed by immunoblotting.

### Co-immunoprecipitation analysis

HeLa cells were transfected with 2.5 μg each of vectors expressing untagged or C-terminally Myc tagged A3G proteins in various combinations. Cells were lysed in 600 ml lysis buffer (0.5% Triton X-100, 287 mM NaCl, 2.68 mM KCl, 1.47 mM KH_2_PO_4_, Na_2_HPO_4_, pH 7.2) as described [32]. Lysates were immunoprecipitated with a Myc-specific monoclonal antibody (clone 9E10; Sigma-Aldrich, Inc., St. Louis MO; Cat # M 4439) as described above. Immunoprecipitates were subjected to immunoblot analysis using an A3G-specific rabbit polyclonal antibody (Apo-C17).

### Membrane floatation analysis (raft association)

Raft association of A3G was assessed by membrane floatation analyses essentially as described by Ono et al [37]. HeLa cells were transfected with 5 μg of wild type and mutant A3G expression constructs DNA (pcDNA-APO3G, pcDNA-APO3G-Myc, pcDNA-APO3G-W127A, pcDNA-APO3G-W127A-Myc, and pcDNA-APO3G-C100S-Myc, respectively). Cells were harvested 20 h later by scraping and washed three times with ice-cold PBS. Cells were pelleted (2,000 × g for 2 min) and resuspended in 300 μl of 10 mM Tris-HCl pH 7.5 supplemented with 4 mM EDTA and Complete™ protease inhibitor cocktail (Roche Diagnostics Corp., Indianapolis IN). After 10 min incubation on ice cells were sonicated for 10 sec and centrifuged for 3 min at 2,000 × g at 4°C in a microcentrifuge to remove insoluble material and nuclei. The postnuclear supernatants (120 μl) were mixed with 120 μl of TNE lysis buffer (100 mM Tris-HCl, 600 mM NaCl and 16 mM EDTA) containing 0.5% Triton X-100 and incubated on ice for 20 min. A total of 200 μl of each lysate was mixed with 1 ml of 85.5% sucrose (w/v) in TNE lysis buffer, placed at the bottom of ultracentrifuge tubes, and overlaid with 2.5 ml of 65% (w/v) sucrose and 1.5 ml of 10% sucrose (w/v) in TNE lysis buffer. The samples were centrifuged at 4°C in a SW55 rotor for 16 hours at 35,000 rpm to obtain Triton X-100 resistant and sensitive fractions. Ten equal fractions (500 μl each) were collected from the top, mixed with 4× sample buffer (180 μl) and boiled. Samples were analyzed by immunoblotting.

### RNA extraction

Total cellular RNA was extracted from untransfected and transfected HeLa cells using the RNeasy RNA extraction kit (QIAGEN, Valencia CA) following the manufacturer's instructions. To isolate RNA form immune complexes, beads were washed three times with RNA-protein binding buffer (20 mM HEPES, 25 mM KCl, 7 mM 2-Mercaptoehanol, 5% Glycerol and 0.1% NP-40). RNA was then extracted using the RNeasy RNA extraction kit. For isolation of genomic RNA precipitated with the A3G complex, *vif*-defective HIV-1 proviral vector DNA (1 μg) was co-transfected into HeLa cells with A3G vectors (4 μg) as indicated in the text. RNA was then extracted from the immunocomplexes as above.

### QRT-PCR

qRT-PCR was performed using the one tube SYBR green method as per manufacturer instruction (AB Biosystems, Warrington UK). Briefly, each 16 μl reaction mixture contained 0.08 μl of Reverse Transcriptase, 0.04 μl of RNase inhibitor, 300 μM of forward and 50 μM of reverse specific primers, 8 μl of 2× SYBR green PCR Master Mix, 2.6 μl of RNase-free water, and 5 μl of template RNA. RNaseA treated RNA from A3G wt samples were used as a negative control. The reactions were performed on a AB Biosystems 7300 Real Time PCR System (AB Biosystems) using the following conditions: 48°C for 30 min followed by 95°C for 10 min and then 40 cycles of 95°C for 15 s and 60°C for 1 min with a dissociation protocol. The target sequences amplified by the SYBR green method used the following primer pairs: 7SL RNA, forward (5'-CCCGGGAGGTCACCATATT-3'), reverse (5'-CTGTAGTCCCAGCTACTCG-3'); HIV-1 genomic RNA, forward (5'TCAGCATTATCAGAAGGAGCCACC-3'), reverse (5'-TCATCCATCCTATTTGTTCCTGAAG-3').

## Results

### Mutation of W127 induces a packaging defect in A3G that is exacerbated by C-terminal epitope tags

**Figure 1:**
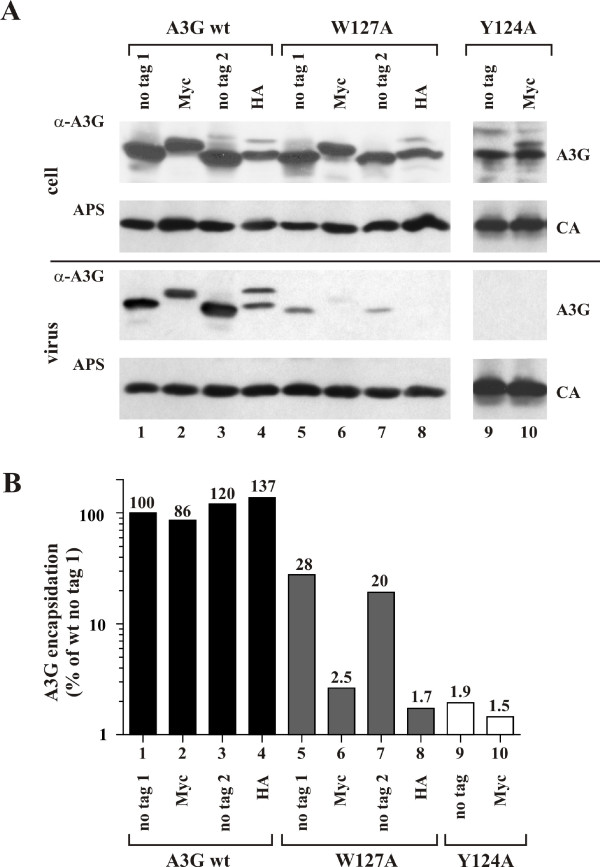
**Expression and packaging of A3G variants into *vif*-deficient HIV-1 virions**. **(A) **HeLa cells were transfected with pcDNA-APO3G (1 μg), pcDNA-APO3G-MycHis (1 μg), pcDNA-APO3G-W127A (1 μg), pcDNA-APO3G-W127A-MycHis (2 μg), pCMV4-APO3G (1 μg), pCMV4-APO3G-HA (2 μg), pCMV4-APO3G-W127A (2 μg), pCMV4-APO3G-W127A-HA (2 μg), pcDNA-APO3G-Y124A (2 μg), and pcDNA-APO3G-Y124A-MycHis (1 μg), together with the *vif*-defective proviral construct pNL43Vif(-) (3 μg). Total amounts of transfected DNA were adjusted to 5 μg using empty vector DNA as appropriate. Cells and virus-containing supernatants were harvested 24 h post transfection and processed for immunoblotting as described in Methods. Blots were probed with antibodies to A3G or an HIV-positive patient serum (APS) to identify viral capsid (CA) protein. Samples in lanes 1 & 3 and 5 & 7 are replicates derived from independent transfections. **(B) **A3G and capsid-specific bands in panel A were quantified by densitometric scanning of the gel and the encapsidation efficiency of A3G was calculated for each variant taking into consideration fluctuations in intracellular A3G expression and viral capsid protein. Results are expressed relative to untagged A3G wt (Fig. 1B, lane 1), which was defined as 100%. Actual values are shown above each column.

Previous work indicated that tagged and untagged variants of wild type A3G (A3G wt) are efficiently incorporated into *vif*-defective HIV-1 virions and exhibit strong antiviral activity. In contrast, HA-tagged A3G W127A and W127L mutants were reported to be packaging incompetent [9,23,26,32]. suggesting that W127 is part of a packaging motif. Similarly, Y124A mutations were found to be poorly packaged [23,26,32]. We first constructed an untagged W127A mutant to study the mechanism of A3G encapsidation into HIV-1 virions. Viral encapsidation of this mutant was compared to untagged A3G wt by coexpression in HeLa cells together with *vif*-deficient NL4-3. Cells and virus containing supernatants from transfected cultures were prepared for immunoblot analysis as described in Methods and probed with antibodies to A3G and an HIV-1-positive patient serum (Fig. 1A). A3G and capsid (CA)-specific bands were quantified by optical scanning and the encapsidation efficiency of A3G was calculated taking into consideration fluctuations in intracellular A3G expression and viral capsid protein (Fig. 1B). Results are expressed relative to untagged A3G wt (Fig. 1B, lane 1), which was defined as 100%. We found that packaging of untagged A3G W127A was reduced 3-5-fold relative to untagged A3G wt (Fig. 1, compare lanes 1 & 3 to lanes 5 & 7.). However, the effect of the W127A mutation on packaging appeared to be relatively modest when compared to previously published data, which showed a much stronger effect [9,23,26,32].

To address potential effects of epitope tags on packaging of A3G, we performed a side-by-side comparison of untagged and epitope tagged A3G wt, A3G W127A, as well as A3G Y124A. For the Y124A mutant the presence or absence of a C-terminal Myc tag was investigated; A3G W127A constructs encoding either a C-terminal Myc tag or an HA tag were analyzed. Amounts of transfected A3G vectors were adjusted as described in the legend to figure 1 to minimize differences in expression or stability of the proteins (Fig. 1A, cell). All DNAs were cotransfected into HeLa cells together with an equal amount of *vif*-defective proviral DNA (pNL4-3Vif(-)) and total amounts of transfected DNA were kept constant. Surprisingly, epitope-tagged A3G W127A variants were much less efficiently packaged into virions than their untagged counterparts (Fig. 1B, compare lanes 5 & 7 to lanes 6 & 8). In contrast, A3G Y124A was packaging incompetent irrespective of the present or absence of a C-terminal Myc tag (Fig. 1B, lanes 9 & 10). Thus, the presence of a C-terminal epitope tag, irrespective of its nature (i.e Myc versus HA), reduced the packaging efficiency of W127A mutants by 40- to 60-fold when compared to untagged A3G wt.

**Figure 2:**
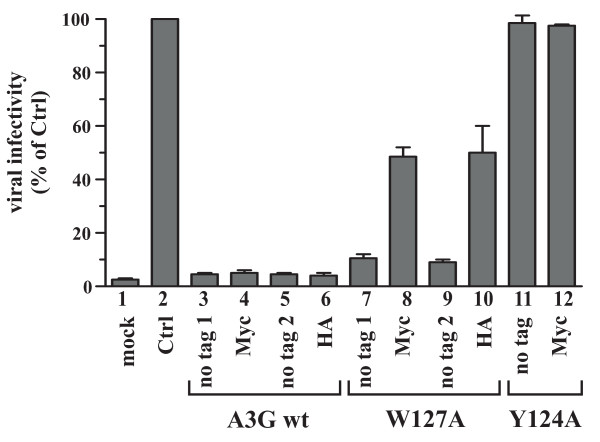
**Incorporation of APO3G inversely correlates with viral infectivity**. Cell free virus particles from figure 1 were normalized for reverse transcriptase activity and used to infect LuSIV indicator cells. Virus-induced activation of luciferase was determined 24 h later in a standard luciferase assay as described in Methods. Mock transfected cells were included as a negative control (mock). Vif-defective virus produced in the absence of A3G served as positive control (Ctrl) and was defined as 100%. Infectivities of the A3G-containing virus preparations were calculated relative to the A3G-negative virus. Error bars reflect standard error calculated from duplicate infections.

Consistent with their poor packaging efficiency, tagged and untagged Y124A mutants as well as epitope tagged A3G W127A mutants exhibited only modest antiviral activity in the context of Vif-defective viruses (Fig. 2, lanes 8, 10, 11 & 12), while untagged A3G W127A strongly inhibited viral infectivity (Fig. 2, lanes 7 & 9). As expected, viruses produced in the presence of A3G wt were non-infectious, irrespective of the presence or absence of a C-terminal epitope tag (Fig. 2, lanes 3-6). These results suggest that Y124 is critical for A3G packaging while the importance of W127 in A3G for virus encapsidation is strongly influenced by the presence or absence of a C-terminal epitope tag.

### Packaging of A3G correlates with lipid raft association

**Figure 3:**
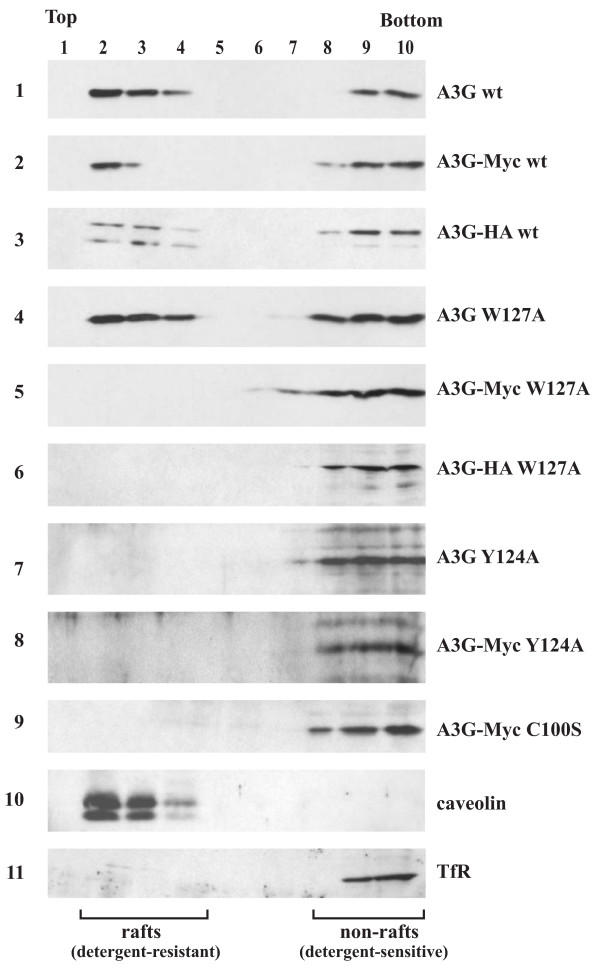
**Floatation analysis of A3G**. HeLa cells were transfected with vectors encoding untagged and C-terminally Myc- or HA-tagged A3G wt (panels 1 - 3), A3G W127A (panels 4 - 6), or A3G Y124 (panels 7-8). The packaging incompetent A3G C100S-Myc variant was included for comparison (panel 9). Cellular caveolin was used as a raft marker (panel 10) and transferrin receptor (TfR) was included as a non-raft associated control (panel 11). Samples were processed for floatation analysis as described in Methods and 10 equal fractions were collected from the top of the gradient. The position of raft and non-raft proteins is indicated at the bottom. Proteins are identified on the right.

A3G was reported to associate with lipid rafts, presumably on intracellular membranes [14]. Since lipid rafts are important for HIV-1 assembly and release [37] we investigated a possible correlation between lipid raft-association and packaging competence of A3G. For this purpose, HeLa cells were transfected with A3G expression vectors encoding untagged and epitope-tagged A3G wt, Y124A, and W127A mutants (Fig. 3). In addition, we included the A3G-Myc C100S mutant as an independent control since it was previously found to be poorly packaged into HIV-1 virions [22]. Cells were harvested 20 h after transfection and processed for floatation analysis as described in the Methods section. As judged from the migration of the lipid raft marker protein caveolin, detergent-insensitive raft-associated proteins were enriched in fractions 2-4 of our floatation gradient (Fig. 3, panel 10). Soluble, detergent-sensitive proteins remained at the bottom of the gradient (fractions 9-10) as exemplified by the migration of transferrin receptor protein (Fig. 3, panel 11 TfR). We found that A3G wt associated with lipid rafts irrespective of the presence or absence of a C-terminal epitope tag (Fig. 3, panels 1 - 3); however, not all of the protein was detergent resistant, consistent with a previous report [14]. Similarly, untagged A3G W127A partitioned with lipid rafts (Fig. 3, panel 4). Interestingly, all five packaging-defective A3G variants, i.e. A3G-Myc W127A, A3G-HA W127A, A3G Y124A, A3G-Myc Y124A, and A3G-Myc C100S, failed to associate with lipid rafts (Fig. 3, panels 5 - 8). Thus, 4 out of 4 packaging competent A3G variants associated with lipid rafts while 5 out of 5 packaging-incompetent variants failed to associate with lipid rafts. These results establish a strong correlation between lipid raft association and packaging competence of A3G.

### A3G packaging competence correlates with the ability to interact with viral genomic RNA

**Figure 4:**
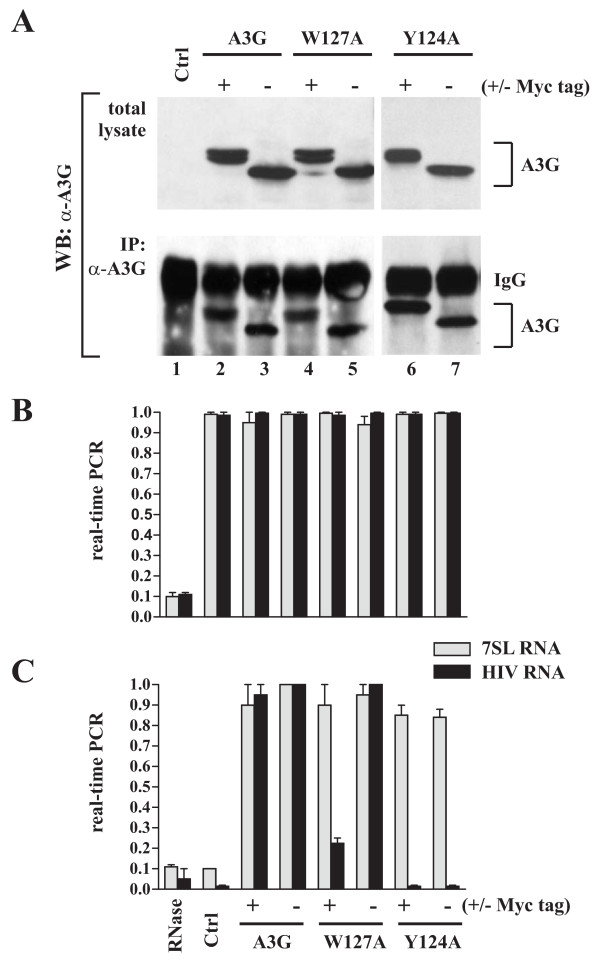
**Packaging incompetent A3G variants are defective for binding viral RNA**. **(A-C) **HeLa cells were transfected with 4 μg of empty vector DNA (lane 1), 2 μg of either Myc-tagged or untagged A3G wt (lanes 2 & 3, respectively), 4 μg of A3G-Myc W127A (lane 4), 2 μg of A3G W127A (lane 5), 2 μg of Y124A (lane 6), or 3 μg of A3G-Myc Y124A (lane 7). All samples were co-transfected with 1 μg of *vif*-defective pNL4-3Vif(-) as a source of genomic RNA. Total amounts of DNA were adjusted to 5 μg using empty vector DNA as appropriate. Cells were harvested 24 h after transfection and divided into four fractions. Fraction 1 was used for immunoblot analysis of whole cell extracts (panel A, top); fraction 2 was used for total RNA extraction and qRT-PCR (panel B). Fractions 3 & 4 were first immunoprecipitated as a pool with an A3G-specific rabbit antibody as described in Methods. Part of the immunoprecipitate (fraction 3) was then used for immunoblotting (panel A, bottom); the other part (fraction 4) was used for RNA extraction and qRT-PCR. **(A) **Fractions 1 & 3 were analyzed by immunoblotting for the presence of A3G using an A3G specific rabbit polyclonal antibody. Proteins are identified on the right. IgG = rabbit immunoglobulin heavy chain. Total cellular RNA **(B) **or RNA present in the immune complexes **(C) **was extracted and subjected to qRT-PCR analysis of 7SL and genomic RNA as described in Methods. 7SL and genomic RNA levels detected in the presence of untagged A3G wt were used as reference and defined as 1.0. RNA levels from all other samples were calculated relative to the reference sample. An RNA sample treated with DNase-free RNaseA (1 mg/ml; 30 min, 37°C) was used as a control for the absence of contaminating DNA.

Previous reports suggested that A3G packaging into HIV-1 virions requires interaction with RNA [7-10,24,32,38,39]. However, there was some discussion about the nature of the RNA mediating A3G encapsidation. One study reported that the packaging defect of A3G W127A was caused by a lack of interaction with 7SL RNA [9]. Other reports including our own argued against a role of 7SL RNA in the packaging of A3G and identified viral genomic RNA as a critical cofactor for A3G encapsidation [8,24]. To assess the importance of W127 and Y124 for interaction with 7SL RNA and/or genomic RNA, we performed a pull-down experiment to identify 7SL RNA and genomic RNA in immunocomplexes of A3G. The impact of a C-terminal epitope tag was assessed using Myc-tagged A3G variants. A3G variants were individually transfected into HeLa cells together with pNL4-3vif(-) DNA as a source of genomic RNA. Transfection conditions were adjusted to ascertain equal expression of all four A3G variants. Cell lysates were prepared 24 h after transfection and used for immunoprecipitation with an A3G-specific antibody. Input material (total cell lysates) and immunoprecipitates were subsequently subjected to immunoblot analysis using an A3G-specific antibody (Fig. 4A, top panel). A sample lacking A3G was included as control (Fig. 4A, lane 1). All A3G variants were expressed at similar levels (Fig. 4A, lanes 2-7) and were precipitated by the A3G-specific antibody with similar efficiency (Fig. 4A, lower panel). Equal samples of the cell lysates and of the immunoprecipitates were used for extraction of total RNA. 7SL RNA and genomic RNA levels in total lysates (Fig. 4B) or in immunoprecipitates (Fig. 4C) were determined by qRT-PCR as described in Methods. As expected, amplification of input samples resulted in very similar signals for 7SL RNA and genomic RNA in all samples (Fig. 4B). Immunoprecipitation of the A3G-deficient sample with the A3G-specific antibody neither pulled down 7SL RNA nor genomic RNA, attesting to the specificity of the immunoselection (Fig. 4C, Ctrl). A3G wt interacted with both 7SL and viral genomic RNA and this interaction was independent of the presence or absence of an epitope tag (Fig. 4C, A3G +/-). Importantly, treatment of A3G wt samples with RNaseA abolished amplification of 7SL and genomic RNA attesting to the absence of contaminating DNA in the RNA preparations (Fig. 4C, RNase). All four A3G variants including A3G-Myc W127A immunoprecipitated similar levels of 7SL RNA (Fig. 4C, W127A & Y124A, grey bars). In contrast, only untagged A3G W127A precipitated wild type levels of viral RNA (Fig. 4C, W127A (-)Myc, black bar) while packaging incompetent A3G variants were severely compromised in their ability to bind viral genomic RNA. These results suggest that viral genomic RNA selectively associates with packaging competent A3G, which is consistent with our previous observations on the importance of viral genomic RNA in the packaging of A3G [7,8].

### Lack of correlation between A3G oligomerization and packaging competence

We previously reported that mutation of C97 in the N-terminal enzymatically inactive deaminase domain of A3G affected oligomerization of the protein but did not abolish packaging or antiviral activity [22]. In contrast, a more recent study concluded that RNA-dependent oligomerization of A3G is required for packaging and for restriction of HIV-1 [32]. This conclusion is based on the observation that the packaging defective A3G Y124 and W127 mutants failed to interact with A3G wt in co-immunoprecipitation studies.

**Figure 5:**
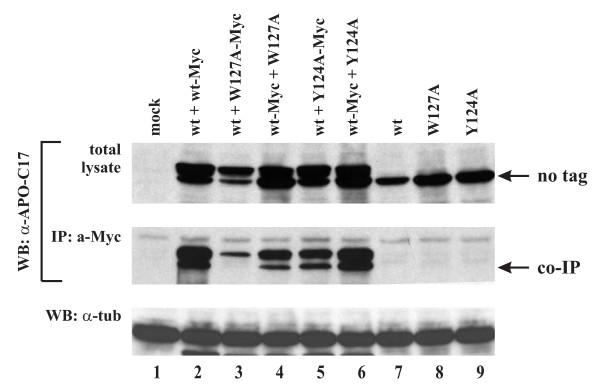
**Lack of correlation of A3G oligomerization and packaging competence**. HeLa cells were transfected with 2.5 μg each of plasmids expressing A3G wt (wt), W127A, or Y124A mutants in a combination of two as indicated above the figure. Cell lysates were analyzed either directly by immunoblotting with antibodies to A3G (top panel) or tubulin (bottom panel) or subsequent to immunoprecipitation with a Myc-specific monoclonal antibody (middle panel). Untagged A3G variants (no tag) have a faster mobility in the gel than the Myc-tagged variants. The position of the untagged proteins co-immunoprecipitated by the Myc-tagged variants is indicated on the right (co-IP).

Since our own packaging studies revealed an impact of C-terminal epitope tags for packaging of A3G W127A, we decided to analyze the correlation between A3G dimerization and packaging competence. To that end we cotransfected A3G wt, A3G W127A, or A3G Y124A mutants in various combinations using untagged or Myc-tagged wild type or mutant A3G constructs. Proteins were immunoprecipitated by a Myc-specific monoclonal antibody followed by immunoblotting with a polyclonal A3G-specific antibody (Fig. 5, middle panel). Total lysates were also probed with an A3G-specific antibody as an input control (Fig. 5, top panel). A tubulin blot was included as loading control (Fig. 5, lower panel). As expected, untagged A3G proteins were not precipitated in the absence of Myc-tagged A3G (Fig. 5, lanes 7-9). Consistent with our previous report [22]. A3G wt interacted with A3G-Myc wt to form homo-oligomers (Fig. 5, lane 2). On the other hand, A3G wt did not seem to interact well with Myc-tagged A3G W127A (Fig. 5, lane 3). However, the reverse combination, i.e. A3G-Myc wt plus untagged A3G W127A, revealed significant interaction (Fig. 5, lane 4). Importantly, the severely packaging impaired Y124A mutants exhibited strong interaction with A3G wt irrespective of which partner in the pull-down assay was tagged (Fig. 5, lanes 5-6). Thus, our data suggest that mutation of W127 and Y124 does not prohibit A3G oligomerization. Therefore, we failed to observe a correlation between A3G oligomerization and packaging competence.

## Discussion

The mechanism of A3G encapsidation into HIV-1 virions has attracted significant attention since it offers a potential target for therapeutic interference with virus replication. There is increasing evidence that encapsidation of A3G into virions requires interactions with the viral nucleocapsid domain in the Gag precursor and involves RNA although the nature of the RNA involved in A3G packaging, i.e. cellular versus viral, remains under investigation [8,9,12-15,18-20,38,41-43]. There is only limited information concerning sequences in A3G that are necessary and sufficient for viral encapsidation. Several studies implicated the N-terminal catalytic domain (CD1) in A3G in RNA interaction and virus encapsidation [5,21,22]. Other studies found that sequences in the N-terminal linker domain downstream of the CD1 domain encompassing residues 122 to 127 were critical for A3G packaging [9,12,20,23,25,26]. However, it remains unclear whether this latter domain represents a direct contact point for protein-protein or protein-RNA interactions or simply represents a conformationally sensitive area in the protein.

Our current study focused on two amino acid residue, W127 and Y124, in A3G to demonstrate that the requirements for A3G packaging are complex and can be influenced by the presence or absence of terminal epitope tags. Our finding that untagged A3G W127A was packaged 10 times more efficient than Myc- or HA-tagged A3G W127A was surprising and unexpected since the interfering epitope tags were not located adjacent to residue W127 but were more than 250 amino acids away at the C-terminus of the protein. There is currently no structure of full-length A3G available. However, computer modelling suggests that W127 is located at the surface of the protein [31]. It is therefore possible that in 3-dimensional space the C-terminus of A3G is in close proximity to the N-terminal linker region surrounding residue W127. Therefore, changes in this region could, in the context of an epitope-tag, induce changes in the protein resulting in mislocalization in the cell - as evidenced by differential raft association - and culminating in the exclusion of A3G from virions. Importantly, the epitope tag effect was not tag specific since identical results were obtained with C-terminal Myc and HA tags. Based on these data we consider it possible that W127 in A3G is critical for proper protein folding/conformation and/or cellular localization of the protein rather than representing a motif required for viral encapsidation.

## Conclusion

Our data reveal an interesting correlation between A3G's propensity to associate with lipid raft structures, viral RNA interaction, and packaging competence. The reason for the inability of the epitope tagged A3G W127A mutants as well as tagged and untagged Y124A mutants to associate with lipid rafts and with viral RNA remains to be investigated. Since A3G is not a membrane protein it is likely that its affinity to raft structures is due to an interaction with other raft associated host factors. Our floatation studies were done in the absence of viral proteins, ruling out the possible contribution of Gag protein in the raft targeting of A3G. Thus, the lack of raft association could be indicative of altered intracellular localization or trafficking of A3G or could be due to a conformational change resulting in the loss of protein-protein interaction.

## Competing interests

The authors declare that they have no competing interests.

## Authors' contributions

MAK conceived the study, performed the molecular and biochemical studies, and drafted the manuscript. RG, EM, and RCW assisted with biochemical studies and helped with data analysis. SK assisted with A3G mutagenesis and biochemical analyses. KS coordinated and supervised the project and wrote the final manuscript.
